# 

**Published:** 1883-10

**Authors:** 


ESTABLISHED IN 1855.

OLD SERIES. Vol. XXI—No. 7. | OCTOBER, 1883. | NEW SERIES Vol. III—No. 1

### ATLANTA MEDICAL REGISTER.

*(ATLANTA MEDICAL AND SURGICAL JOURNAL.)*

EDITED BY

J. H. LOGAN, A.M., M.D.

vol. 3
1883-84.

H. H. DICKSON, PUBLISHER. TERMS, $1.50 A YEAR IN ADVANCE.

SURGEON GEN'L'S OFFICE
LIBRARY.
94433

ATLANTA, GEORGIA:
*H. H. DICKSON, BOOK AND JOB PRINTER AND BOOKBINDER.*
1883. -84.

